# The Value of Electroretinography in Identifying Candidate Genes for Inherited Retinal Dystrophies: A Diagnostic Guide

**DOI:** 10.3390/diagnostics13193041

**Published:** 2023-09-25

**Authors:** Tsai-Hsuan Yang, Eugene Yu-Chuan Kang, Pei-Hsuan Lin, Pei-Liang Wu, Jacob Aaron Sachs, Nan-Kai Wang

**Affiliations:** 1 Department of Education, Chang Gung Memorial Hospital, Linkou Medical Center, Taoyuan 33305, Taiwan; yangth@cgmh.org.tw; 2 College of Medicine, National Yang Ming Chiao Tung University, Taipei 11217, Taiwan; 3 Department of Ophthalmology, Chang Gung Memorial Hospital, Linkou Medical Center, Taoyuan 33305, Taiwan; yckang@cgmh.org.tw; 4 College of Medicine, Chang Gung University, Taoyuan 33302, Taiwan; 5 Graduate Institute of Clinical Medical Sciences, College of Medicine, Chang Gung University, Taoyuan 33302, Taiwan; 6 National Taiwan University Hospital, Yunlin 640203, Taiwan; y04507@ms1.ylh.gov.tw; 7 Department of Ophthalmology, Edward S. Harkness Eye Institute, Columbia University Irving Medical Center, Columbia University, New York, NY 10032, USA; b10401094@ntu.edu.tw (P.-L.W.); jas1068@miami.edu (J.A.S.); 8 Department of Medicine, National Taiwan University, Taipei 10617, Taiwan; 9 College of Arts and Sciences, University of Miami, Coral Gables, FL 33146, USA; 10 Vagelos College of Physicians and Surgeons, Columbia University, New York, NY 10032, USA

**Keywords:** electroretinography, inherited retinal dystrophies, electronegative ERG, congenital stationary night blindness, X-linked retinoschisis, cone–rod dystrophies, fundus albipunctatus, enhanced S-cone syndrome, cone dystrophy with supernormal rod response

## Abstract

Inherited retinal dystrophies (IRDs) are a group of heterogeneous diseases caused by genetic mutations that specifically affect the function of the rod, cone, or bipolar cells in the retina. Electroretinography (ERG) is a diagnostic tool that measures the electrical activity of the retina in response to light stimuli, and it can help to determine the function of these cells. A normal ERG response consists of two waves, the a-wave and the b-wave, which reflect the activity of the photoreceptor cells and the bipolar and Muller cells, respectively. Despite the growing availability of next-generation sequencing (NGS) technology, identifying the precise genetic mutation causing an IRD can be challenging and costly. However, certain types of IRDs present with unique ERG features that can help guide genetic testing. By combining these ERG findings with other clinical information, such as on family history and retinal imaging, physicians can effectively narrow down the list of candidate genes to be sequenced, thereby reducing the cost of genetic testing. This review article focuses on certain types of IRDs with unique ERG features. We will discuss the pathophysiology and clinical presentation of, and ERG findings on, these disorders, emphasizing the unique role ERG plays in their diagnosis and genetic testing.

## 1. Introduction

### 1.1. Background and Significance of Electroretinography (ERG)

Inherited retinal dystrophies (IRDs) stem from genetic mutations. In addition to clinical and imaging assessments, identifying these mutations is crucial for a definitive diagnosis, which can lead to improved disease classification and pathophysiology research.

Though genome sequencing costs are decreasing rapidly [1], using multimodal ophthalmic examinations to narrow the differential diagnosis and sequence only the disease-associated genes is still preferred. Sequencing a patient’s entire genome is utilized less due to certain limitations. Firstly, false positive signals can be generated, and interpreting them can be challenging. Secondly, incidentally found genetic mutations that have no onset or have low penetrance are possible. Deciding whether or not to inform a patient of these mutations requires careful ethical consideration, well-established policies, and an evaluation of the cost-effectiveness and benefits of any further preventative measures [2,3].

Therefore, narrowing the differential diagnosis before ordering disease-specific candidate gene sequencing is crucial. To achieve this, it is necessary to look into the personal and family history of the patient and conduct multiple ophthalmic examinations that complement each other’s diagnostic limitations. Diseases of the inner retina may be associated with a normal fundus appearance. While optical coherence tomography (OCT) is a valuable tool in detecting structural abnormalities in different retina layers, functional retinal dysfunction requires electrophysiological examinations for proper diagnosis. Full-field ERG (ffERG) distinguishes between generalized outer or inner retinal abnormalities and determines whether the dysfunction is predominantly in the bipolar cells or photoreceptors (rod or cone cells).

This review aims to offer guidance on using ffERG to pinpoint potential genes for sequencing, leading to a conclusive diagnosis. We cover the physiological basis of ERG to aid in understanding the origin and formation of ERG features in IRDs. Our focus is on monogenic IRDs that display distinct ERG patterns, where we discuss the pathophysiology, clinical presentation, ERG features, and genes responsible for these diseases.

### 1.2. Anatomy and Physiology of the Retina

The vertebrate retina consists of ten anatomical layers. The retinal pigmented epithelial cells (RPE) provide support for the neural retina, which is mainly composed of three types of neurons connected in the following order: photoreceptors, bipolar cells, and ganglion cells.

**Figure 1:**
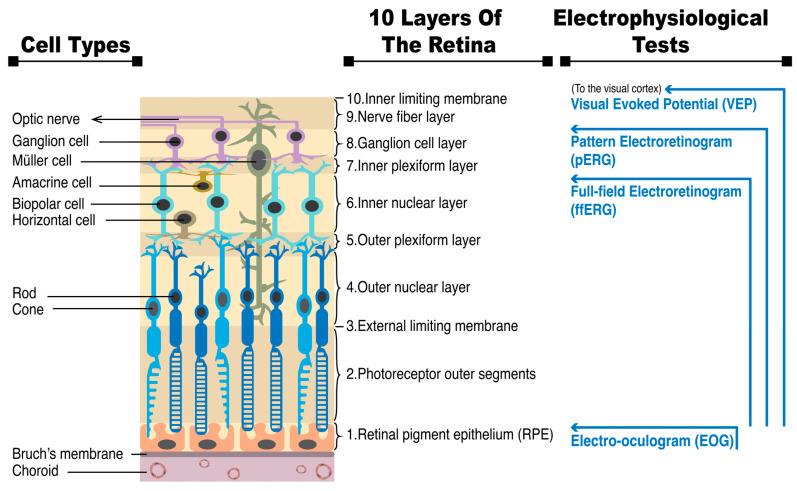
The retina anatomy and various electrophysiological tests gathering collective cellular reactions from multiple levels of the retina.

The retina’s photoreceptor layer comprises rods and cones with different visual functions, distributions, and connections to post-synaptic bipolar cells. The outer segments of photoreceptors contain photopigments consisting of chromophore 11-*cis*-retinal and opsins. Rhodopsins are the photopigments in rods that provide monochromatic vision. Rods are sensitive to single protons, making them important for visual perception in dim light. Conversely, cones function in well-lit conditions and are responsible for color perception through short, middle, and long opsins. Rods are more densely packed than cones throughout most of the retina, except in the fovea, where cones are most abundant and contribute to high visual acuity in that area [4]. Rods connect to On-bipolar cells, while cones synapse to On- or Off-bipolar cells. Acknowledging the different functions, distributions, and connections to post-synaptic bipolar cells of rods and cones can aid in comprehending the various symptoms of rod- or cone-dominant IRDs and help in understanding the formation of ERG. Figure 1 displays the retina anatomy and various electrophysiological tests that gather collective cellular reactions from multiple levels of the retina. Further elaboration on these topics will be provided below.

The visual pathway is such that light stimulation on the retina leads to visual perception in the brain. This process begins with phototransduction, where light stimulates photoreceptors. The next stage, a post-receptoral process within the retina, involves bipolar and ganglion cell activation. Finally, the signal transmits beyond the retina, through the lateral geniculate nucleus, and to the brain’s visual cortex, forming a visual perception. The initial two stages of the visual process, which occur in the retina, will be covered here.

**Figure 2:**
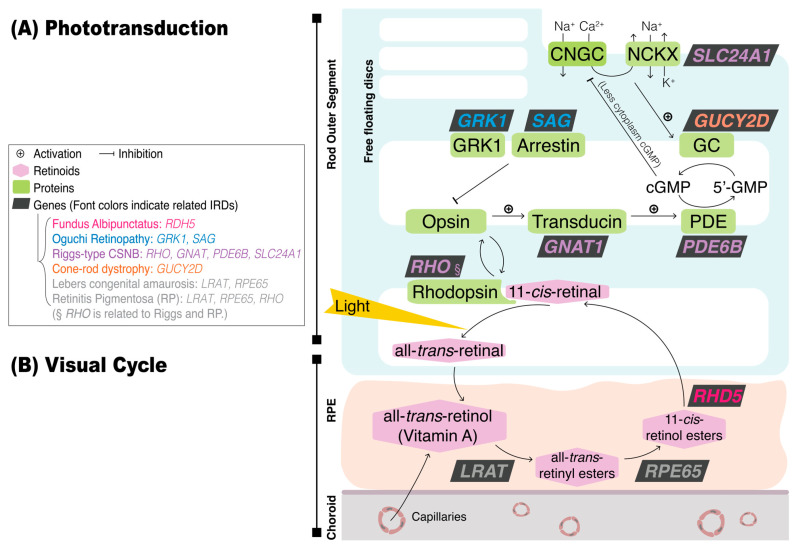
The schematic of genes and encoded proteins involved in phototransduction and the visual cycle. Genes in different colored fonts indicate the responsibility for various inherited retinal diseases (IRDs): orange for fundus albipunctatus, blue for Oguchi retinopathy, green for Riggs-type congenital stationary night blindness (CSNB), purple for *GUCY2D*-related cone–rod dystrophy, and grey for Leber congenital amaurosis or retinitis pigmentosa (RP). Genes in the graph are specifically expressed in rods, except for *GUCY2D,* expressed in both rods and cones. The diagram shows the process in rods, but the proteins used in cones are the same, except that rhodopsin should be replaced with cone-opsins. (**A**) *Phototransduction.* Rhodopsin is located in the outer segments of rods and consists of 11-cis-retinal chromophore and opsin. To begin, light stimulates 11-cis-retinal to isomerize into all-trans-retinal and changes the shape of opsins, activating transducin, the G-protein coupled to rhodopsin. This, in turn, activates photoreceptor phosphodiesterase (PDE), which hydrolyzes cyclic guanosine monophosphate (cGMP) and leads to the closure of the cyclic nucleotide-gated ion channel (CNGC). Therefore, photoreceptors are hyperpolarized, reducing glutamate release to bipolar cells. The recovery of phototransduction involves two parts: first, rhodopsin activity is terminated by rhodopsin kinase (GRK1) and arrestin; second, calcium efflux through sodium/calcium–potassium exchanger (NCKX) activates guanylyl cyclase (GC), which replenishes cGMP. (**B**) *Visual cycle.* To maintain phototransduction processing, the visual cycle recycles retinoids between the photoreceptors and RPE to replenish 11-cis-retinal in the rhodopsin. Key enzymes involved in this process include lecithin retinol acyl transferase (LRAT), RPE65 protein, and 11-cis-retinol dehydrogenase (RDH), which are encoded by genes responsible for IRDs—*LRAT*, *RPE65*, and *RDH5*, respectively. The process requires all-trans-retinol, or vitamin A, to be repleted from choroid capillaries.

The process of phototransduction in photoreceptors converts photons into electrophysiological signals by bleaching photopigments, which requires the visual cycle between photoreceptors and RPE cells to replenish the photopigments [5]. Phototransduction begins when light stimulates 11-cis-retinal to isomerize into all-trans-retinal. This triggers conformational changes in opsins, interrupting cyclic guanosine monophosphate (cGMP) accumulation and leading to the closure of the constantly open cation channels. This, in turn, causes the photoreceptors to become hyperpolarized, decreasing the levels of glutamate released from the photoreceptors to the post-synaptic bipolar cells. To maintain phototransduction processing, the visual cycle recycles retinoids between the photoreceptors and RPE to replenish the 11-cis-retinal in the rhodopsin. Figure 2 depicts the genes and encoded proteins involved in phototransduction and the visual cycle. Mutations in these genes can cause IRDs such as fundus albipunctatus, Oguchi retinopathy, Riggs-type CSNB, or *GUCY2D*-related cone–rod dystrophy.

A post-receptoral process in the visual pathway occurs following phototransduction in the photoreceptors. When the photoreceptors decrease glutamate release upon exposure to light, On-bipolar cells (which respond to the onset of light) depolarize, while Off-bipolar cells (which respond to the offset of light) hyperpolarize. As a result, post-synaptic ganglion cells generate action potentials projecting beyond the retina through the lateral geniculate nucleus and ultimately reach the visual cortex in the brain [6].

In the following section, we will explore how ERG can aid in identifying issues in phototransduction or post-receptoral processes within the retina.

### 1.3. Principles of ERG Recording and Methodological Details

Electrophysiological examinations are essential in diagnosing the functional integrity of the visual system. As displayed in Figure 1, various tests assess different parts of the visual system, such as the visual evoked potentials (VEPs) for the whole visual pathway from the eye to the visual cortex of the brain, electro-oculogram (EOG) for the outer retina and RPE, pattern ERG (pERG) to differentiate between the functions of macular and retinal ganglion cells, and multifocal ERG (mfERG) for testing multiple discrete areas of the retina simultaneously [7,8,9,10]. This article specifically focuses on the use of ffERG in diagnosing IRDs. This test measures the massed electrical response of the retina to light stimuli, allowing for the assessment of generalized retinal function involving the rod, cone, and bipolar cells.

To ensure consistency in recording ERG tests, the International Society for Clinical Electrophysiology of Vision (ISCEV) has established standards [11]. This facilitates cross-laboratory comparisons and is crucial for research on retinal diseases and accurate patient monitoring over time. Electrodes are attached to the cornea, bulbar conjunctiva, or skin of the lower eyelids of an electrically isolated patient according to the ISCEV standard protocol to measure the retina’s response to flashes from a Ganzfeld stimulator, which evenly illuminate the entire retina. The protocol can begin with either dark-adapted (DA), also referred to as scotopic, ERG or light-adapted (LA), also called photopic, ERG. After 20 min of dark adaptation, the retina’s response to flashes of varying strengths (0.01, 3, and 10 cd·s·m^−2^) can be evaluated to assess rod-dominated retinal function (DA 0.01 ERG; DA 3 ERG; DA 10 ERG). Alternatively, a 10 min period of light adaptation can reduce rod involvement and increase the cone system’s selective contributions, allowing for the recording of the retina’s response to single flashes (LA 3 ERG) or high-frequency flashes (LA 30 Hz ERG) of 3 cd·s·m^−2^ illuminance that are superimposed on a light-adapting background. The flash durations should be at least 5 milliseconds each, and the inter-stimulus interval should be at least 2 s for DA 0.01 ERG, 10 s for DA 3 ERG, 20 s for DA 10 ERG, and 0.5 s for LA 3 ERG, allowing all components of ERG waveforms to be recorded. At least 20 ms of baseline waveform before stimuli should be included for artifact calibration.

To assist with diagnosing certain disorders, standard ERG recordings may be supplemented with additional recordings. For instance, On–Off ERG uses extended photopic stimulation to separate the On-bipolar response to light onset and the Off-bipolar response to light offset while S-cone ERG measures the specific response of S-cones using short wavelength flashes superimposed on a background that adapts the rods and L/M-cones [12,13].

### 1.4. ERG Waveforms and Components

In order to localize the dysfunctional site of the retina, it is essential to know the origin of the ERG components. As previously discussed, light stimulation hyperpolarizes photoreceptors, leading the bipolar cells to depolarize. This process can be recorded in the ERG waveform, where a negative “a-wave” originates from photoreceptor hyperpolarization and a positive “b-wave” signifies bipolar cell depolarization, but only if the photoreceptors are functional.

**Figure 3:**
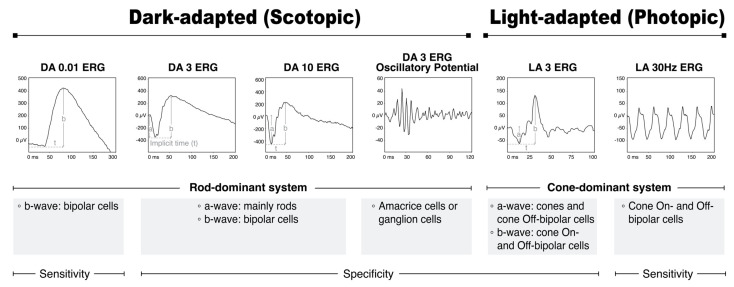
The reference ranges of standard full-field ERG (ffERG) according to the ISCEV protocol. The diagram illustrates the measurement of the amplitudes (solid vertical lines) and implicit times (broken horizontal lines marked as “t”) of the standard ERG components, which consist of a-waves and b-waves of dark-adapted (DA) and light-adapted (LA) single-flash responses. Moreover, the origins of the a-waves and b-waves in retinal cells are shown.

Figure 3 shows the reference ranges of the ISCEV’s standard ffERG protocols [7]. In a scotopic setting, a dim flash, not bright enough for DA 0.01 ERG to record the response from rods but bright enough to record the amplified response from the bipolar cells, generates a b-wave reflecting the rod system sensitivity [8]. Stronger flashes produce a mixed rod–cone response that is dominated by the rod system, eliciting DA 3 ERG and DA 10 ERG. Additionally, oscillatory potentials observed in the rising limb of b-waves may indicate activity in amacrine cells and retinal ganglion cells. In patients with opaque media, small pupils, or immature retinae, DA 10 ERG may be a valuable clinical tool.

Alternatively, in a photopic setting where the response of the rods is saturated, LA 3 ERG and LA 30 Hz ERG reflect the cone system function. LA 3 ERG consists of an a-wave from cones and cone Off-bipolar cells and a b-wave from both the On- and Off- bipolar cells. In addition, LA 30 Hz ERG is generated by the On- and Off-bipolar cells.

Regional retinal dysfunction results in a decreased amplitude in the a- or b-wave. This is measured by looking at the baseline-to-trough region for a-waves and the trough-to-peak region for b-waves. On the other hand, a more widespread dysfunction of the retina may cause delayed “implicit time”, or the time between the light stimuli and the b-wave peak.

### 1.5. Electronegative ERG: Pathophysiology and Associated Diseases

An electronegative waveform, also known as a waveform from negative ERG, is characterized by a smaller b-wave than the normal-sized a-wave [9]. This differs from healthy individuals, where the b-wave is larger than the a-wave. This pattern can be observed in DA 3 ERG, DA 10 ERG, or LA 3 ERG, and it pinpoints the location of retinal dysfunction as being post-receptoral, including bipolar cells or the synapse between photoreceptor and bipolar cells. Identifying this pattern can help narrow down the genes likely associated with these IRDs.

It is important to note that electronegative ERG patterns are not exclusive to IRDs [10]. They may also manifest in acquired conditions that impact one or both eyes, including but not limited to siderosis, drug toxicity, central retinal artery occlusion (CRAO), central retinal artery occlusion (CRVO), paraneoplastic or autoimmune retinopathies, and vitamin A deficiency [11,12,13]. It is helpful to gather information about a disease’s onset time, progression course, and family history to better understand and differentiate between inherited and acquired diseases.

Here, we delve into three distinct categories that classically manifest electronegative ERG: congenital stationary night blindness (CSNB), X-linked retinoschisis (XLRS), and cone dystrophy due to *CRX* gene mutation. ERG plays a crucial role in the differential diagnosis of these conditions in order to test genes for mutations.

## 2. Congenital Stationary Night Blindness (CSNB): Riggs, Complete (cCSNB), and Incomplete (iCSNB)

### 2.1. Pathophysiology

There are four types of CSNBs, namely Riggs, Schubert–Bornschein, fundus albipunctatus, and Oguchi disease [14]. The Schubert–Bornschein type can be further categorized into complete (cCSNB) and incomplete (iCSNB) forms. This section will cover Riggs-type CSNB, cCSNB, and iCSNB, as they display distinctive ERG patterns, while the topic of fundus albipunctatus will be addressed in a separate section.

Various types of CSNB originate from genetic mutations of the different retinal cells. Riggs-type CSNB occurs due to genetic mutations that encode proteins involving rod phototransduction, leading to abnormal rods. Meanwhile, iCSNB and cCSNB are both caused by dysfunctional bipolar cells. However, iCSNB affects both On- and Off-bipolar cells due to abnormal synapses between photoreceptors and bipolar cells, while in cCSNB, the patient selectively loses the response of On-bipolar cells. Specifically, as the locus of *CACNA1F* is within the photoreceptor pre-synaptic membrane involving the release of glutamate release, gene mutations of iCSNB cause both ON and OFF pathways to be affected [15]. The genetic mutations that cause cCSNB include *GRM6*, which produces glutamate receptors on the On-bipolar cells, and *TPRM1*, which generates ion channels that depolarize On-bipolar cells. Additionally, there are proteins that interact with *TPRM1* channels, including nyctalopin encoded by *NYX* and proteins expressed by *LRIT3* [16,17,18,19,20].

### 2.2. Clinical Presentation

Patients with CSNB commonly experience night blindness, which may be accompanied by myopia, strabismus, or nystagmus. Night blindness, or nyctalopia, typically begins before adulthood and does not progress over time, although some cases have been reported where symptoms do progress or nyctalopia is absent [21]. The presence of strabismus and nystagmus suggests that there has been a sub-optimal visual perception in the brain since the development of the visual cortex during infancy [22,23]. Patients with autosomal recessive cCSNB and *NYX* mutation often have high myopia, while those with autosomal recessive iCSNB and *CACNA1F* mutation may develop myopia or hyperopia. On the other hand, those with autosomal dominant Riggs and *RHO* mutation do not typically have myopia, strabismus, or amblyopia [24,25].

Family history, fundus imaging, and ERG are necessary tools to distinguish the different types of CSNB, which further indicate the genes to test for confirmative diagnosis. First, the types of CSNBs can be distinguished based on inheritance patterns: autosomal dominant (AD), autosomal recessive (AR), or X-linked recessive. Second, the fundus imaging of fundus albipunctatus shows small white dots scattered across the posterior pole, except on the fovea, while Oguchi disease displays a gray-white metallic sheen that disappears after dark adaptation, known as the Mizuo–Nakamura phenomenon. These fundus appearances are unique and distinguishable from Riggs-type CSNB, cCSNB, and iCSNB, which have normal fundus appearances. Lastly, their ERG patterns can differentiate cCSNB, iCSNB, and Riggs. Table 1 categorizes the types of CSNB according to fundus appearance, inheritance pattern, and dysfunctional retinal cells and lists the reported gene mutations.

### 2.3. ERG Findings and Candidate Genes

ERG is crucial in distinguishing the three types of CSNB with normal fundus appearance: Riggs-type CSNB, cCSNB, and iCSNB. To start, Riggs-type CSNB is identified by a flat scotopic a-wave. Next, to differentiate between cCSNB and iCSNB, which both typically display electronegative ERG reports although these are not necessary for diagnosis, photopic ERG is used.

Riggs-type CSNB has defective rods and normal cones, resulting in a flat wave in DA 3 ERG and normal photopic responses. On the other hand, iCSNB and cCSNB have post-receptoral bipolar cells that are affected but also have intact photoreceptors, causing electronegative DA ERG reports and abnormal photopic responses [26].

Photopic ERG can help distinguish between iCSNB and cCSNB. First, iCSNB has a more reduced b-wave in LA 3 ERG, consistent with abnormal synapses between cones and both On- and Off-bipolar cells. The 30 Hz flicker amplitude is severely subnormal and may display notched, or bifid, peaks [9]. Second, an LA ERG report of cCSNB has a preserved b-wave, in line with preserved Off-bipolar function. The a- and b-waves have unique shapes: the b-wave has a sharp rise and is without oscillatory potentials while the a-wave is normal with a wide trough.

If additional On–Off ERG tests are performed, the findings would be consistent with the pathophysiology: iCSNB would exhibit reduced rapid On and Off responses, while cCSNB would display a reduced rapid On response but intact rapid Off response.

**Figure 4:**
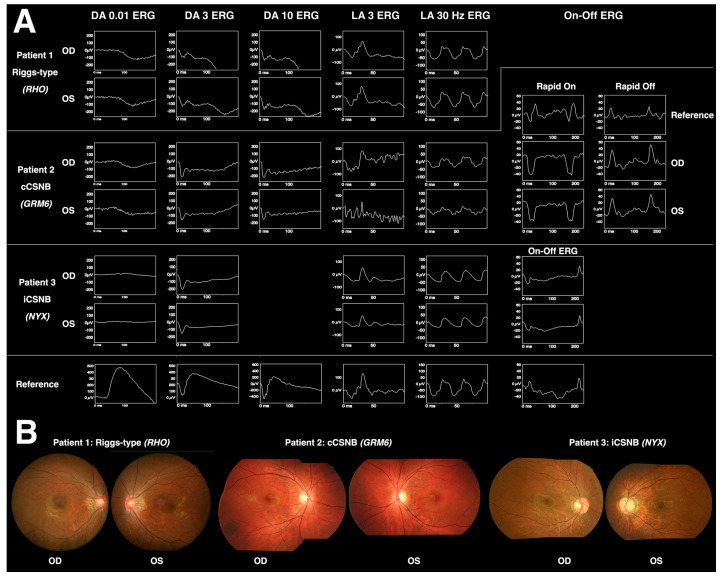
The fundus and ERG images of Riggs-type CSNB, cCSNB, and iCSNB in a Taiwanese cohort. Patient 1 has *RHO*-mutated Riggs-type CSNB, patient 2 has *GRM6*-mutated cCSNB, and patient 3 has *NYX*-mutated iCSNB. Panel (**A**) shows the ERG patterns, while panel (**B**) shows the fundus examination results. These patients present with similar clinical features and imaging, while the distinctive ERG patterns play a pivotal role in identifying candidate genes for definitive diagnosis [21].

The ERG findings for Riggs-type CSNB, iCSNB, and cCSBN are summarized in Table 2. Figure 4 displays a Taiwanese cohort’s fundus and ERG findings for Riggs-type CSNB, cCSNB, and iCSNB. The patients in the figure present with similar clinical features and imaging, while the distinctive ERG patterns play a pivotal role in identifying candidate genes for definitive diagnosis.

### 2.4. Differential Diagnosis, Treatments, and Prognosis

Combining Table 1 with Table 2, which outlines the ERG characteristics for these diseases, can assist in diagnosing a patient who displays symptoms of congenital stationary night blindness.

The ERG patterns of CSNBs can be indistinguishable from those of certain acquired retinal diseases. For example, the ERG pattern of cCSNB is similar to that of melanoma-associated retinopathy (MAR) due to the presence of autoantibodies against *TRPM1* [27]. This is also a gene mutation responsible for cCSNB. Another example is that the ERG patterns of fundus albipunctatus and retinopathy are associated with vitamin A deficiency, which is comparable. Both conditions result from dysfunction in the retinoid cycle. Proper diagnosis requires a thorough investigation of the patient’s family and personal history, including the onset of visual symptoms, diet, pregnancy, and any history or symptoms of cancer.

*RHO* mutations can lead to not only Riggs-type CSNB but also retinitis pigmentosa (RP). Despite both diseases being inherited in an AD pattern and presenting nyctalopia, they differ in clinical course and ERG results. *RHO*-related CSNB typically does not progress in symptoms, whereas *RHO*-related RP does. In cases of RHO-related CSNB, the DA 0.01 ERG report shows a flat a-wave, while the DA 3 or DA 10 ERG reports show reduced a-waves and negative b-waves. However, photopic responses remain intact, indicating that only the rods are affected. Conversely, in typical cases of *RHO*-related RP, scotopic and photopic responses are extinguished [21,28].

Several cases of CSNB are reported to be progressive, emphasizing the need for therapeutic efforts in CSNB [29,30]. Animal trials show structural recovery in *NYX*, *LRIT*, and *GRM6* knockout by adeno-associated virus (AAV)-mediated gene therapy [20,31,32,33,34]. However, unlike the success in treating *RPE65* mutations in Leber Congenital Amaurosis [35], there have not been successful human clinical trials for treating CSNB. This may be due to the fact that amblyopia and nystagmus, common symptoms of CSNB, develop during the development of the infant’s visual cortex. Therefore, it is most efficient to reverse these symptoms during the early childhood stage [36]. To accurately evaluate the benefits of clinical trials targeting amblyopia or nystagmus, younger patients within the optimal therapeutic window should be considered.

## 3. X-Linked Retinoschisis (XLRS)

### 3.1. Pathophysiology

The cause of XLRS is a mutation in the *RS1* gene that produces the retinoschisin protein responsible for the adhesion and interaction between photoreceptors and bipolar cells [37]. When this protein is dysfunctional, it results in schisis in the neural retina.

### 3.2. Clinical Presentation

XLRS patients experience early-onset visual loss. The fundus imaging shows cystic changes in the macula, or the typical spoke-wheel-like pattern, with or without peripheral schisis. OCT can detect cystoid macular edema (CME) in the neural retina [38,39]. Nowadays, although fluorescein angiography (FA) is not required to diagnose XLRS, it confirms the CME to be non-leaking, which can also be observed in other conditions such as nicotinic acid toxicity, enhanced S-cone syndrome, and optic pit-related macular edema [40].

There is a wide range of phenotypes in XLRS patients, which may differ significantly among or within families with the same variants. In younger patients, the spoke-wheel pattern in the fundus imaging may be obscured by conditions such as vitreous hemorrhage, retinal detachment, and leukocoria mimicking Coat-like disease [40,41,42,43]. It is important for clinicians to consider the possibility of XLRS in young patients who present with unsatisfactory BCVA along with vitreous hemorrhage or leukocoria. In older patients, macula schisis may resolve, leaving atrophic changes.

### 3.3. ERG Findings and Diagnostic Criteria

**Figure 5:**
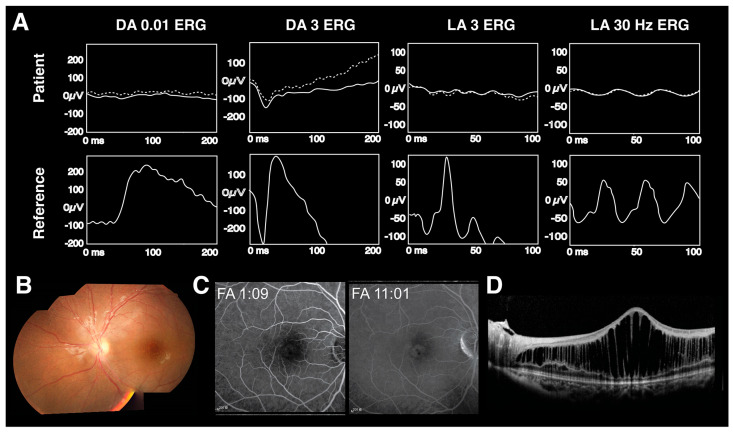
The typical findings from X-linked retinoschisis (XLRS) in a Taiwanese cohort [40]. (**A**) ERG results revealing electronegative DA 3 ERG and reduced scotopic response. (**B**) Spoke-wheel-like pattern at the macula. (**C**) Fluorescein angiography (FA) shows non-leaking cystoid macular edema (CME). (**D**) Schisis and increased thickness at the macula.

XLRS patients have dysfunctional synapses between photoreceptors and bipolar cells, resulting in both the typical electronegative findings in DA 3 ERG as well as reduced scotopic response. However, it is important to note that not all patients with XLRS will have an electronegative scotopic ERG report, and a normal b/a ratio in their DA test results does not necessarily rule out the diagnosis of XLRS [40]. Figure 5 displays typical fundus, FA, OCT, and ERG findings in a Taiwanese cohort of XLRS patients [40].

### 3.4. Management and Prognosis

In terms of managing XLRS patients, there are positive findings from the use of carbonic anhydrase inhibitors (CAIs) and preimplantation genetic diagnosis to conceive a sibling of an XLRS patient [40], but gene therapies have not shown promising results.

CAIs have been used to treat schisis or CME, which is achieved by modulating carbonic anhydrase IV receptors on the RPE membrane or by enhancing fluid transport [44,45]. However, the effectiveness of CAIs varies, and reports suggest that their impact is more visible in improving central foveal thickness and less so in improving visual acuity [40,46,47].

The eye is an ideal target for gene delivery systems to restore functional retinal proteins because of its accessibility and immune-privileged status [48]. However, while the AAV vector-based gene therapy drug was successful in treating patients with confirmed biallelic *RPE65* mutation-associated Leber Congenital Amaurosis (LCA) and was approved by the FDA in 2017 [35], clinical trials of gene therapy on XLRS patients have shown unpromising results. There have been two clinical trials for gene therapies administered through intravitreal injection and mediated by AAV, of which one trial (ClinicalTrials.gov: NCT02416622) was stopped due to a high number of inflammatory adverse events, such as vitritis, while the other (ClinicalTrials.gov: NCT02317887) did not show significant improvements in a preliminary report [49]. A new technique has been developed to enhance previous trials’ effectiveness called “AAV.SPR”, and this uses AAV capsids that can spread beyond the injection site under the retina, ensuring the safe delivery of the *RS1* gene to photoreceptors in the central retina/foveal region [50]. The United States Food and Drug Administration (FDA) has recently approved a clinical trial for XLRS patients that will utilize the AAV.SPR delivery system and be administered through a subretinal injection (ClinicalTrials.gov: NCT05878860) [51].

To conclude, in order to investigate the accurate efficacy of CAIs or *RS1*-gene therapy on XLRS, larger prospective randomized trials involving younger participants with less severe retinal structural changes are necessary.

## 4. *CRX*-Related Cone–Rod Dystrophy

### 4.1. Pathophysiology

The *CRX* gene is located in the cone–rod homeobox gene, involving the development and maintenance of photoreceptors [52]. Pathogenic mutations in the *CRX* gene may give rise to a wide spectrum of retinal diseases including progressive cone–rod dystrophy [53]. Although the retinal expression of *CRX* is limited to photoreceptors, this dysfunction may result from faulty photoreceptor communication with second-order retinal neurons [54].

### 4.2. Clinical Presentation

Patients with CRX-related progressive cone–rod dystrophy inherit in an AD manner, with onsets occurring at adulthood [55]. The phenotype and penetrance widely vary [56]. Since the cones are more affected than rods, patients present with difficulty in visual acuity, central vision, color discrimination, or photophobia and show maculopathy in fundus imaging. As the disease progresses, rod dysfunction becomes more apparent, leading to loss of the peripheral visual field and nyctalopia.

### 4.3. ERG Findings

**Figure 6:**
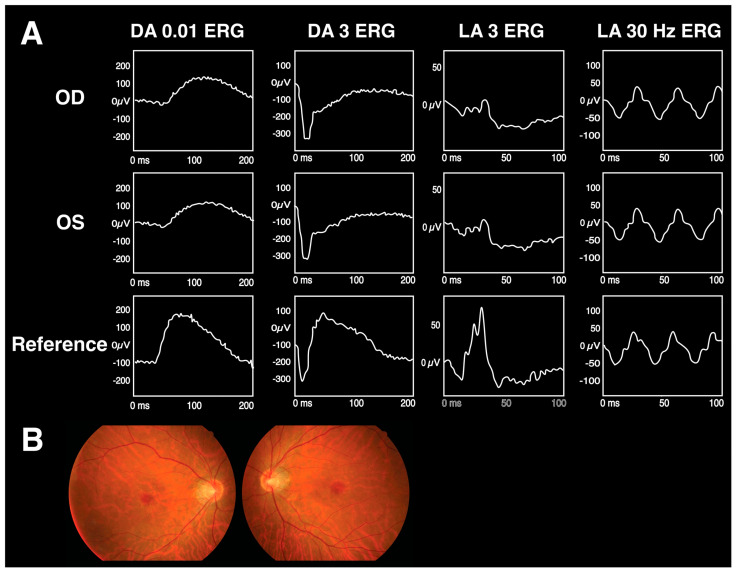
The findings on *CXR*-related cone–rod dystrophy in a Taiwanese case. (**A**) ERG report revealing reduced scotopic response and electronegative DA 3 ERG. (**B**) Fundus examination.

In addition to the ERG pattern of cone–rod dystrophy, where photopic ERG responses are more affected than scotopic responses, patients with *CRX*-related cone–rod dystrophy typically present with electronegative waveforms in DA 3 or 10 ERG tests, consistent with the dysfunction site between photoreceptors and second-order retinal neurons [57]. The electronegative ERG feature is a highly sensitive diagnostic marker in patients with *CRX*-related cone–rod dystrophy [57]. It helps rule out CRX retinopathy in family members, as this may be the only clinical abnormality detected in asymptomatic patients [58]. Figure 6 shows the fundus and ERG pattern in a case of *CRX*-related cone–rod dystrophy.

### 4.4. Other Genes with These Features

Similar to the *CRX* mutation, there are other gene mutations that cause AD-inherited progressive cone–rod dystrophy and show the electronegative scotopic response to strong light stimuli, including the *RAX2, GUCY2D,* and *PRPH2* genes.

The *RAX2* gene, previously named *QRX* or *RAXL1*, has a sequence homologous to the *CRX* gene wherein the two genes encode proteins to interact synergistically to be involved in transcription in the retina’s outer and inner nuclear layers [59]. Mutations in the *GYCY2D* gene cause defects in photoreceptor synaptic transmission to second-order neurons, consistent with the electronegative waveform [60]. *PRPH2* mutations can give rise to not only AD-inherited progressive cone–rod dystrophy but also macula dystrophy and rod-cone dystrophy [61,62]. The gene encodes peripherin, a transmembrane protein in the outer segments of the photoreceptors, and contributes to the site’s morphogenesis, stabilization, and renewal [55,56].

## 5. Fundus Albipunctatus

### 5.1. Pathophysiology

Fundus albipunctatus, an autosomal recessive disease, is caused by mutations in the *RDH5* gene, which encodes retinol dehydrogenase 5, an enzyme expressed at RPE [63]. It involves the visual cycle, which oxidates 11-cis-retinol into 11-cis-retinal, to help recycle the chromophore back to its original form before receiving light stimuli. The interruption of this process restricts the photopigment turnover and leads to reduced lipofuscin formation, which is derived from the photoreceptor outer segments debris. The process is displayed in Figure 2.

### 5.2. Clinical Presentation

Fundus albipunctatus is classified under the disease category of CSNB due to its characteristic of non-progressive night blindness. Distinguishing this disease from other types of CSNB involves observing abnormal findings in the fundus and noting the improvement of scotopic ERG responses after an extended period of dark adaptation due to the photopigment’s slow regeneration rate.

As the name suggests, fundus imaging shows retinal white-yellowish dots at the posterior pole, sparing the fovea. Using fundus autofluorescence (FAF), lipofuscin accumulation in the patient’s RPE can be mapped in vivo [64], and this shows hypo-FAF signals [65]. Infrared scanning laser ophthalmoscopy (IR) provides detailed images of subretinal lesions and displays hyperreflective spots [66].

### 5.3. ERG Findings

**Figure 7:**
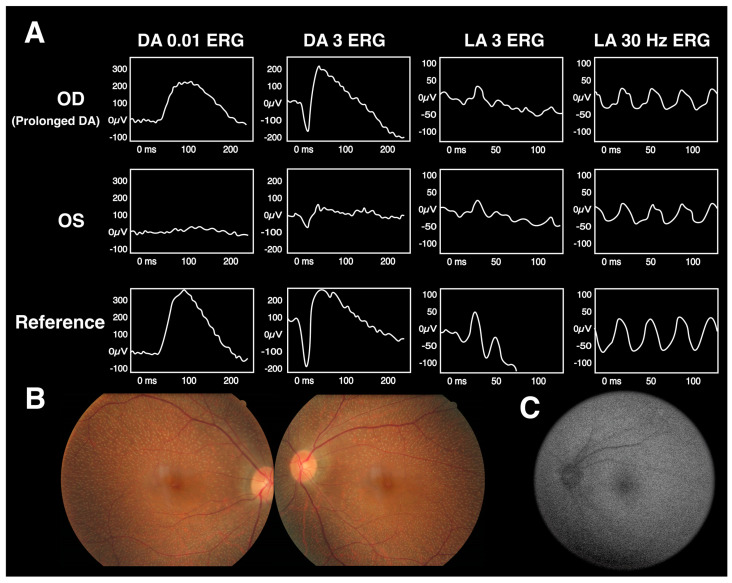
The findings on fundus albipunctatus in a Taiwanese case [66]. (**A**) ERG results revealing recovery of the DA 3 ERG response after prolonged scotopic adaptation. (**B**) Fundus examination with retinal white dots at the posterior pole, sparing the fovea. (**C**) Fundus autofluorescence (FAF) showing mottling hypo-autofluorescent signals.

The ERG patterns of fundus albipunctatus are characteristic of the recovery of the scotopic response after prolonged scotopic adaptation [67]. Electronegative ERG results in response to a bright flash may occur due to the dark-adapted cone system being exposed without a normal rod function [65]. They should not be assumed to reflect inner retinal rod system dysfunction. Figure 7 depicts the fundus imaging, FAF, and ERG examinations of a patient with fundus albipunctatus.

### 5.4. Differential Diagnosis

In addition to being found in patients with fundus albipunctatus, white retinal dots can also be observed in those with retinitis punctata albescens (RPA) [68] caused by *RLBP1* mutations and in cases of benign retinal flecks. However, the absence of any abnormalities in ERG can differentiate benign retinal flecks from other conditions. To distinguish between RPA and fundus albipunctatus, it is noteworthy that abnormal ERG patterns in RPA do not improve after extended scotopic adaptation, and the symptoms of nyctalopia and restricted visual field worsen over time [69].

## 6. Enhanced S-Cone Syndrome (ESCS), or Goldmann–Favre Syndrome

### 6.1. Pathophysiology

Enhanced S-cone syndrome (ESCS), also known as Goldmann–Favre syndrome, is an autosomal recessively inherited retinal dystrophy caused by pathogenic mutations in the orphan nuclear receptor transcription factor (*NR2E3*) gene, which is responsible for the suppression of cone-specific genes in rods during embryogenesis [70]. *NR2E3* mutations result in excess S-cones, and the decreased number of rods in and clumping of S-cones lead to retinal dysplasia and degeneration.

### 6.2. Clinical Presentation

Patients with ESCS present with nyctalopia, variable visual acuity loss, and constricted field of vision [71]. The fundus in ESCS patients may show round pigment clumping along the vascular arcades in the mid-periphery of the retina in older patients or retinal white dots in younger patients [72,73,74]. OCT reveals cystoid macular edema [75,76], which can be confirmed to be non-leaking by using FA. The rosette-like lesions from the RPE in OCT correspond to hyper-autofluorescent signals resulting from resident microglia infiltration [77,78].

### 6.3. ERG Findings

**Figure 8:**
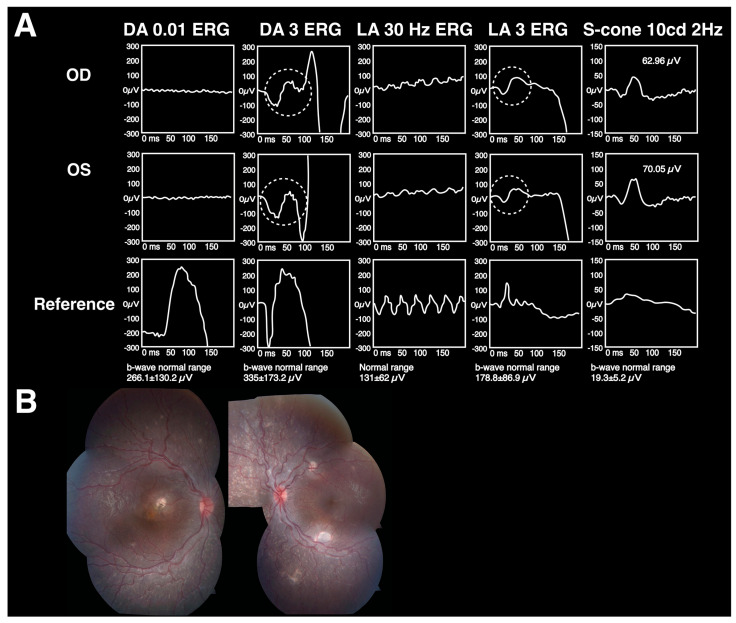
The findings on a patient with Enhanced S-cone syndrome (ESCS), also known as Goldmann–Favre syndrome [77]. (**A**) Three distinct ERG features, including extinguished DA 0.1 ERG, similar waveforms in DA 3 or 10 ERG reports and LA 3 ERG, and a larger a-wave in LA 3 ERG than in LA 30 Hz ERG. Moreover, excess S-cones can be verified by observing an elevated signal in S-cone ERG. (**B**) Fundus examination revealing retinal white dots.

Patients with ESCS exhibit three distinct ERG features. Firstly, the cone-dominated retina causes the DA 0.01 ERG results to be extinguished. Secondly, waveforms in DA 3 or 10 ERG reports are similar to those in LA 3 ERG in shape as both are light-stimulated responses from mere cones, not rods. Thirdly, the LA 3 ERG a-wave shows increased amplitudes due to having an increased number of cones, with amplitudes larger than those of the LA 30 Hz ERG wave. Moreover, the presence of excess S-cones can be verified by observing an elevated signal in S-cone ERG. Figure 8 demonstrates the fundus and ERG findings on ESCS patients.

### 6.4. Differential Diagnosis

Both ESCS and RP can manifest with pigmentary changes in the fundus. To differentiate, the pigmentary changes in ESCS are round and distributed in the mid-periphery of the retina, while those of RP appear as bone spicules [79,80].

## 7. Cone Dystrophy with Supernormal Rod Response (CDSRR)

### 7.1. Pathophysiology

Cone dystrophy with supernormal rod response (CDSRR) is an autosomal recessively inherited retinal dystrophy caused by pathogenic mutations in the potassium voltage-gated channel modifier subfamily V member 2 (*KCNV2*) gene. This gene is expressed in rods and cones and is responsible for the assembly of potassium voltage-gated channels [81].

### 7.2. Clinical Presentation

Dysfunctional cones cause reduced central visual acuity and color vision while abnormal rods lead to photophobia and nyctalopia [82]. These symptoms typically appear within the first two decades of life [83]. Fundus imaging is limited in its ability to diagnose CDSRR due to the possibility of non-specific appearances including maculopathy. Instead, a CDSRR diagnosis is based on identifying distinctive ERG findings due to *KCNV2* mutations.

### 7.3. ERG Findings

Patients with CDSRR have two distinguishable features. Firstly, the photopic response is reduced and delayed to a great extent, consistent with universal cone dysfunction. Secondly, DA 0.01 ERG is reduced and significantly delayed while DA 3.0 ERG showed a disproportionately augmented b-wave, or a “supernormal rod response” manifests. Testing all patients with these two characteristic ERG features is recommended since the *KCNV2* gene has two exons, making direct sequencing affordable [84].

### 7.4. Differential Diagnosis

**Figure 9:**
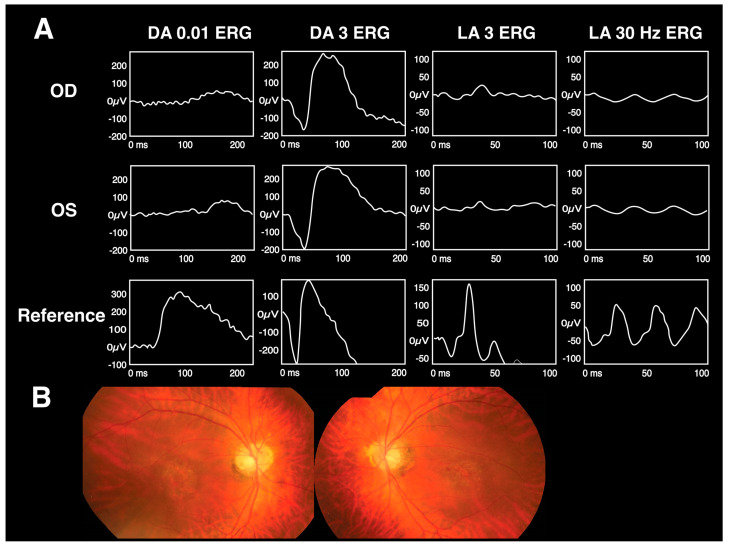
The findings on a patient with cone dystrophy with supernormal rod response (CDSRR), who was initially mistaken as having HCQ retinopathy [84]. The distinct ERG patterns helped pinpoint the candidate gene for a definite diagnosis. (**A**) ERG report showing a supernormal rod response to bright flash intensity in the setting of cone and rod dystrophy. (**B**) Fundus imaging showing symmetric atrophic lesion at the central macula in both eyes.

In patients with hydroxychloroquine (HCQ) retinopathy, the toxicity initially affects the photoreceptor and then progresses to the RPE cells. The bull’s eye maculopathy in the fundus imaging of CDSRR patients may be mistaken as HCQ retinopathy, but OCT, FAF, and ERG can help distinguish the two causes [85]. It should be noted that a history of taking HCQ does not rule out the differential diagnosis of CDSRR [84]. Figure 9 displays the fundus and ERG findings on a patient with CDSRR, initially mistaken for HCQ retinopathy.

In severe cases of HCQ retinopathy, OCT may exhibit a flying saucer sign, indicating the loss of parafoveal structure [86]. Additionally, FAF may display parafoveal hyper-autofluorescent with or without parafoveal dark areas [87]. While ERG patterns may vary, they do not exhibit supernormal rod response to bright stimuli in the presence of reduced cone and rod response.

## 8. Challenges and Limitations of ERG in Diagnosis

### 8.1. Technical Considerations

In clinical settings where IRDs are being investigated, mydriasis or ophthalmic examinations with bright illumination are frequently conducted. There are certain technical concerns to consider if ERG is recorded following these examinations [7].

To begin with, mydriasis is not mandatory as per the latest ISCEV-standard ERG protocol as long as the reference ranges are used with the same settings. Nevertheless, if mydriasis is used, it is necessary to document the pupil diameter before and after ERG recording.

Second, following intensely illuminated ophthalmic examinations, it is essential to recover in a well-lit area for at least 30 min before performing an ERG recording to obtain accurate data.

### 8.2. Variability and Standardization of ERG Protocols

For consistent ERG recording across different subjects, visits, and laboratories, it is essential to standardize ERG protocols based on the ISCEV’s standard protocol. It is recommended to establish reference values specific to each laboratory using subjects with similar demographics to the patient population. ERG for infants under 6 months of age must be approached cautiously as their readings may be unclear or show lower amplitudes and longer peak times [7]. In general, photopic ERG tests in infants mature by 3 months, while scotopic ERG tests mature by 6 months.

The ERG results of patients with refractive errors or comorbidities should be interpreted with caution. In myopic patients, the b-wave amplitudes were found to be lower than in patients without refractive errors, with scotopic responses being more affected than photopic responses. This effect is more pronounced in patients with myopic-related structural retinal changes [88,89,90]. Patients with glaucoma or optic nerve diseases may exhibit reduced b-wave amplitudes in DA 0.01 ERG, but they may have larger amplitudes for the photopic negative response, that is, the negative waveform following the b-wave. However, the diagnostic value of ffERG, which is based on a- and b-waves, remains unaffected and is still crucial in diagnosing various eye diseases.

The laboratory-specific reference values should be included in the ERG reports of patients to facilitate interpretation. Additionally, it is useful to document the testing time, pupil diameters, and the type of the recording electrodes used. Specifically, the type of the recording electrodes is important to note, as contact-lens-recording electrodes tend to show higher ERG wave amplitudes compared to lower-eyelid-skin electrodes [91].

### 8.3. Importance of Integrating Clinical Findings and Multimodal Imaging with ERG

Genetic testing is essential for managing IRD patients. It aids in providing an accurate diagnosis, predicting the prognosis, communicating the risk of inheritance, and determining eligibility for gene-based therapies or clinical trials.

In such cases, targeted sequencing guided by clinical assessment is preferred over whole-genome sequencing (WGS) or whole-exome sequencing (WES) approaches for several reasons [92]. Compared to WGS, targeted sequencing requires less cost, data storage, and effort to analyze incidental findings that are not related to the presenting disease. Alternatively, unlike WES, targeted sequencing is capable of detecting deep intronic mutations, which could be the root causes of certain IRDs [93,94].

Performing the target sequencing of genes related to suspected IRDs requires precise clinical assessment, which highlights the importance of integrating clinical findings, multimodal imaging, and ERG reports. Personal and family medical history should be obtained when evaluating people with suspected IRDs. This should include any symptoms of rod (such as nyctalopia or peripheral visual field loss) or cone dysfunction (such as impaired central or color vision or photophobia), the age at which symptoms began and how they progressed, and any medication usage. Multimodal imaging is then used to evaluate structural changes. To clarify, fundus imaging can distinguish between different types of CSNBs. OCT allows for a cross-sectional view of the retinal layers, such as in the detection of retinal schisis in XLRS patients. Additionally, hypo-autofluorescent signals can indicate lower lipofuscin in the RPE, consistent with fundus albipunctatus. Finally, ERG tests are utilized to identify the specific layer of dysfunctional retinal cells that plays a critical role in differential diagnosis.

## 9. Conclusions

### 9.1. Summary of Findings

IRDs are a clinically and genetically heterogeneous group of diseases with emerging therapeutics signaling the need for precise diagnosis. IRDs primarily affect the rod or cone photoreceptors or inner retinal neuronal layers, where ERG identifies the defective region and guides genetic tests for confirmative diagnosis.

An electronegative waveform in scotopic ERG, which is a lower amplitude of b-wave compared to that of the a-wave, indicates inner retinal diseases. These include complete, incomplete, and Riggs-type CSNB, XLRS, and cone–rod dystrophies related to *CRX, GUCY2D, PRPH2,* or *RAX2*. Electronegative waveforms are not typical of fundus albipunctatus, ESCS, and CDSRR, but they have their own distinctive ERG patterns.

This review provides a guide for diagnosing IRDs that exhibit specific ERG features. Table 2 summarizes the symptoms, multimodal imaging findings, inheritance patterns, and ERG features of these IRDs. This information guides the clinical assessment and further pinpoints the specific genes to be tested for diagnosis.

### 9.2. Clinical Implications and the Unique Role of ERG in Diagnosing Select Retinal Disorders

ERG is essential throughout stages of IRD management, including clinical assessment for guiding genomic tests and tracking visual function over time [95].

Precise clinical assessments of IRDs are important because they are crucial for both disease diagnosis, such as in aiding the interpretation of genetic variants of uncertain significance [96], and for research purposes, like in segregation studies of candidate genetic variants [97]. Precise clinical assessments of IRDs rely on ERG rather than just imaging studies. While fundus imaging is useful for studying anatomical pathologies, stable fixation is necessary for high-quality images [98,99]. Conversely, ERG examines functional dysfunctions in retinal cells that may occur before ophthalmoscopic changes and does not require as much stable fixation during recording as other electrophysiology techniques [7]. These features render ERG a crucial tool for accurately diagnosing IRDs, particularly in younger patients, as young patients with IRDs are more susceptible to the onset of these diseases, may have nystagmus affecting fundus image quality, and may benefit from early interventions.

ERG offers direct retinal response measurements, with a broad application and non-invasive nature, but it is limited by variability and requires further genetic testing for definite diagnosis. In contrast, NGS provides comprehensive gene screening with high sensitivity, but it is cost-intensive and demands complex data interpretation. Microarray analysis, being high-throughput as well but more affordable than NGS, can miss rare variants and hinges on known genetic data. In conclusion, while ERG furnishes essential functional insights, its diagnostic capacity is augmented when paired with molecular techniques like NGS and microarray. This combined approach enriches our understanding and diagnosis of inherited retinal dystrophies.

### 9.3. Limitations and Future Research Advancements in ERG Applications

There are potential limitations that may impede the broad acceptance of ERG in diagnosing IRDs. Advanced ERG devices could be expensive, which might limit their accessibility, especially in resource-limited settings. The specificity and sophistication of ERG demand expertise, which could be a barrier in places with a shortage of trained professionals. Some medications and substances, including alcohol or sedatives, can affect the ERG waveform [100]. A comprehensive medical history and understanding of potential interactions are essential for accurate interpretation.

The technology of ERG has advanced in the areas of portable devices and the use of machine learning for analysis and modeling [101]. Handheld ERG technology provides portable and accessible recording that would help identify IRDs in clinical settings that need further referral to specialized centers and for uncooperative or supine-positioned infants. This technology has produced results consistent with standard systems in pediatric patients [102]. Additionally, reports of handheld ERG devices are being used to diagnose specific IRDs [103].

Artificial intelligence (AI) has been widely applied to healthcare systems, including in the field of ERG. Studies showed that machine learning could help classify ERG features in patients with IRD, other ocular diseases, or psychiatric diseases [104,105,106]. For decades, ERG has played a vital role in studying the retina. Although retinal imaging has improved, ERG remains relevant for quantitatively assessing retinal cells in vivo. With advancing technology and AI-assisted analyses, testing for retinal diseases is becoming more accessible and informative.

## Figures and Tables

**Table 1:** Classification of congenital stationary night blindness (CSNB) and its reported gene mutations by dysfunction site, fundus appearance, and inheritance pattern.

		Congenital Stationary Night Blindness (Mutated Genes)			
**Dysfunctional retinal cells**	**Bipolar cells**	iCSBN (*CACNA1F*) cCSNB (*NYX*)	N/A	iCSBN (*CABP4, CACNA2D4*) cCSNB (*GPR170, GRM6, LRIT3, TRPM1*)	N/A
	**Rods**	N/A	Riggs (*GNAT1, PDE6B, RHO*)	Riggs (*SLC24A1*)	Oguchi (*SAG, GRK1*)
	**RPE**	N/A	N/A	N/A	Fundus albipunctatus (*RDH5*)
**Fundus appearance**		Normal fundus			Abnormal fundus
**Inheritance pattern**		X-linked recessive	Autosomal dominant	Autosomal recessive	

Abbreviations: RPE, retinal pigment epithelium; iCSNB, incomplete congenital stationary night blindness; cCSNB, complete congenital stationary night blindness; N/A, not applicable.

**Table 2:** Summary of inherited retinal dystrophies with distinct electroretinography (ERG) features.

Disease	Ocular/Fundus Abnormalities	Inheritance Patterns: Mutated Genes	Scotopic ERG		Photopic ERG		Comments
			DA 0.01 ERG	DA 3 or 10 ERG	LA 3 ERG	LA 30 Hz	
**1.** **Characteristically Presents with Electronegative DA ERG**							
**Incomplete Congenital Stationary Night Blindness** **(iCSNB)**	- Sx: non-progressive nyctalopia, strabismus, nystagmus - Normal fundus - *CACNA1F* mutation may present myopia or hyperopia	- XR: *CACNA1F* - AR: *CABP4, CACNA2D4*	↓ (“Incompletely” abolished)	↓ b-wave (-Ve, but not required)	↓b-wave	- ↓ amplitude & ↑ implicit time - Bifid peaks	- Affects On- and Off-bipolar cells (∴ -Ve) (∴ LA ERG more affected than cCSNB) - On–Off ERG: both ↓
**Complete Congenital Stationary Night Blindness** **(cCSNB)**	- Sx: same as iCSNB - *NYX* mutations associated with high myopia - Normal fundus	- XR: *NYX* - AR: *GPR17, GRM6, LRIT3, TRPM1*	↓↓ Extinguished (“Completely” abolished)	↓ b-wave (-Ve, but not required)	- Normal a-wave with wide trough - Preserved b-wave (sharp rise without oscillatory potentials)	Normal, possible ↑ implicit time	- Affects On-bipolar cells (∴ -Ve) (∴ Preserved b-wave in LA 3 ERG) - On–Off ERG: On↓, Off preserved - DDx: **MAR** (∵ anti-*TRPM1*)
**Riggs-type CSNB**	- Sx: *RHO* mutations develop nyctalopia, but not myopia, strabismus, or nystagmus - Normal fundus	- AD: *GNAT, PDE6B, RHO* - AR: *SLC24A1*	↓↓ Extinguished	↓ a-wave & ↓ b-wave (Possibly -Ve)	Normal		- Affects rods (∴ ↓ a-wave) - DDx: *RHO* mutation also causes **retinitis pigmentosa** (symptoms progress; both scotopic and photopic extinguished)
**X-linked Retinoschisis** **(XLRS)**	- Fundus: spoke-wheel appearance at the macula; could be obscured by vitreous hemorrhage - OCT: central schisis/CME, with or without peripheral schisis - FA: non-FA leakage CME	XR: *RS1*	↓	↓ b-wave (-Ve, but not required)	↓	↓	- Affects the synapse between photoreceptors and bipolar cells (∵ structural schisis at the neural retina) - Rx: carbonic anhydrase inhibitor (improves schisis structure but not BCVA)
**Cone–rod Dystrophy**	- Sx: ↓visual acuity, central vision, color discrimination - Fundus: maculopathy	AD: *CRX, GUCY2D, PRPH2, RAX2*	↓	↓ b-wave (-Ve)	↓↓		- Cones more affected than rods - Adult onset - Progressive symptoms
**2.** **Not characteristic of electronegative DA ERG**							
**Fundus** **Albipunctatus**	- Sx: Non-progressive nyctalopia improving after prolonged dark adaptation - Fundus: White dots at the posterior pole, sparing the fovea - FAF: hypo-AF signals	AR: *RDH5*	- Recovery of scotopic ERG after prolonged dark adaptation - ↓ a-wave & ↓ b-wave (Possibly -Ve)		Varies		- Affects RPE/visual cycle (∴ recovery of scotopic ERG after prolonged dark adaptation) - DDx: **retinitis punctata albescens** (*RLBP1* gene), **benign retinal flecks**
**Enhanced** **S-cone Syndrome (ESCS)/** **Goldmann-Favre Syndrome**	- Fundus: white dots at young age, round pigment clumps at the mid-periphery - OCT: CME, retinal rosettes - FA: non-FA leakage CME - FAF: hypo-AF signals of microglias	AR: *NR2E3*	↓↓Extinguished (∵ cone-dominated retina)	- Similar waveforms in DA 3 or 10 ERG and LA 3 ERG (∵ cone-dominated retina) - ↑LA 3 ERG a-wave (∵ increased cones), which is larger than LA 30 Hz ERG wave			- ↓ cone function - ↓ rod function (∵ cone-dominated retina) - Triad of distinct ERG features - S-cone ERG: ↑ - DDx: **retinitis pigmentosa** (bone-spicule pigments at the equator)
**Cone Dystrophy with Supernormal Rod Response (CDSRR)**	- Sx: impaired central and color vision, photophobia, nyctalopia - Fundus: maculopathy - FAF: ↓ foveal, ↑parafoveal ring	AR: *KCNV2*	↓ and delayed	↑ b-wave disproportionally	↓↓ (Cones more affected than rods)		- ↓↓ cone function and ↓ rod function - DDx: **hydroxychloroquine retinopathy** (CDSRR should not be excluded in patients taking hydroxychloroquine)

Distinct features for diagnosis are in an underlined font. Abbreviations: DA, dark-adapted; LA, light-adapted; XR, X-linked recessive; AD, autosomal dominant; AR, autosomal recessive; ↓, decreased; ↓↓, decreased more; ↑, increased; -Ve, electronegative ERG; Sx, symptoms; DDx, differential diagnosis; Rx, treatments; BCVA, best corrected visual acuity; MAR, melanoma-associated retinopathy; XLRS, X-linked retinoschisis; FA, fluorescein angiography; AF, auto-flrorescence; OCT, optical coherence tomography; FAF, fundus autofluorescence; CME, cystic macular edema; N/A, not applicable; ∵, because; ∴, therefore.

## Data Availability

Not applicable.
